# Delamination Depth Detection in Composite Plates Using the Lamb Wave Technique Based on Convolutional Neural Networks

**DOI:** 10.3390/s24103118

**Published:** 2024-05-14

**Authors:** Asaad Migot, Ahmed Saaudi, Victor Giurgiutiu

**Affiliations:** 1 Department of Petroleum and Gas Engineering, College of Engineering, University of Thi-Qar, Nasiriyah 64001, Iraq; 2 Department of Communication and Electronics Engineering, College of Engineering, University of AL-Muthanna, Samawah 66001, Iraq; ahmed.saaudi@mu.edu.iq; 3 Department of Mechanical Engineering, University of South Carolina, 300 Main Street, Columbia, SC 29208, USA; victorg@sc.edu

**Keywords:** Lamb waves, CNN, GoogLeNet, composites, delamination, SLDV, wavefield images, wavenumber spectrum

## Abstract

Delamination represents one of the most significant and dangerous damages in composite plates. Recently, many papers have presented the capability of structural health monitoring (SHM) techniques for the investigation of structural delamination with various shapes and thickness depths. However, few studies have been conducted regarding the utilization of convolutional neural network (CNN) methods for automating the non-destructive testing (NDT) techniques database to identify the delamination size and depth. In this paper, an automated system qualified for distinguishing between pristine and damaged structures and classifying three classes of delamination with various depths is presented. This system includes a proposed CNN model and the Lamb wave technique. In this work, a unidirectional composite plate with three samples of delamination inserted at different depths was prepared for numerical and experimental investigations. In the numerical part, the guided wave propagation and interaction with three samples of delamination were studied to observe how the delamination depth can affect the scattered and trapped waves over the delamination region. This numerical study was validated experimentally using an efficient ultrasonic guided waves technique. This technique involved piezoelectric wafer active sensors (PWASs) and a scanning laser Doppler vibrometer (SLDV). Both numerical and experimental studies demonstrate that the delamination depth has a direct effect on the trapped waves’ energy and distribution. Three different datasets were collected from the numerical and experimental studies, involving the numerical wavefield image dataset, experimental wavefield image dataset, and experimental wavenumber spectrum image dataset. These three datasets were used independently with the proposed CNN model to develop a system that can automatically classify four classes (pristine class and three different delamination classes). The results of all three datasets show the capability of the proposed CNN model for predicting the delamination depth with high accuracy. The proposed CNN model results of the three different datasets were validated using the GoogLeNet CNN. The results of both methods show an excellent agreement. The results proved the capability of the wavefield image and wavenumber spectrum datasets to be used as input data to the CNN for the detection of delamination depth.

## 1. Introduction

Structures are frequently subject to damage, which is a major factor in structural failure. Unexpected structural failure can have severe effects on society, in addition to resulting in the loss of life and economic ruin. Early damage detection is essential for avoiding sudden and disastrous structural system failures and breakdowns. The non-destructive testing (NDT) techniques include various methods to estimate the integrity of the tested structure. These NDT techniques can provide sufficient information about the structure’s state and identify the shape, size, and location of various flaws [1]. Structural health monitoring (SHM), a branch of NDT, is applied to evaluate the condition of various structures during the service [2,3]. In recent years, a significant focus has been put on structural damage identification in early conditions to prevent the unexpected breakdown of structural elements. In the fields of civil and mechanical engineering, numerous strategies and tools for investigating structures and recognizing damages have been developed [4,5]. The SHM field is an emerging approach that aims to give an early warning of damage initiation and to identify structural issues like fatigue damage [6,7]. The SHM methodology often entails the installation of sensors to gather important data and provide judgments regarding the dependability and continuing life of the structure [8,9]. The researchers have shown tremendous interest in guided waves, one of the most appealing techniques for health monitoring large structures [10]. Lamb waves can propagate across a considerable distance in metallic and composite materials; as a result, a large region can be promptly investigated [11]. The estimation of delamination size was experimentally examined utilizing Lamb waves, a network of piezoelectric wafer active sensors (PWASs), and a developed imaging technique. It was discovered that this developed methodology can precisely predict the delamination size and its shape [12]. Many projects were taken and put into practice to use the generated scattering-guided Lamb waves to measure structural flaws [13]. Numerous academics have recently investigated the potential of PWASs for SHM purposes such as investigating the integrity of metallic and composite plates and localizing the impacts on aerospace structures. These sensors can be utilized as passive and active sensing devices [14,15,16].

Composite materials are being used extensively in aeronautical industries because of their high specific strength and stiffness [17]. The severity of damage to composite materials is higher than that to metallic ones. There are numerous damage types can exist in composite materials, including fiber breakage, and delamination. The most frequent and risky type of failure for aerospace composite structures is delamination, which develops without any obvious surface damage, is challenging to spot visually, and is difficult to detect efficiently [18,19]. Different techniques, including Lamb wave methods, acoustic emission, and electromechanical impedance measurement have been utilized for the detection and identification of structural delamination [20,21,22]. The Lamb wave modes can investigate the whole laminate structure, allowing for the finding of interior as well as external damage [11]. A scanning laser Doppler vibrometer (SLDV) has recently become commonly used for acquiring precise wavefield data of the scanning area due to its benefits in precise surface velocity measurement over a regionally intensive grid [23]. The analysis of acquiring data from the investigated structure is an essential step for evaluating the structure’s integrity and identifying damages. Several data analysis approaches were found to evaluate the structural defects, including wavefield and wavenumber processing techniques [24,25]. A wavefield imaging approach was used to locate simulated delamination in a composite plate [26].

Artificial intelligence (AI) is a wide-science concept that aims to mimic the human way of thinking by applying AI-based models to machines. The AI simulation techniques involve problem-solving and decision-making tasks [27]. The AI-based techniques are used to develop rule-based, ontology, and inference engines that are used to analyze data and make a decision. SHM techniques are examples of using AI-based systems that read data to make decisions. AI has made a significant impact on fields like voice recognition, autonomous vehicles, precise healthcare, and the identification of many diseases. Its uses for handling enormous amounts of data have been efficiently proved on different systems [27,28,29]. Machine learning (ML) techniques are used in the AI field. ML algorithms help machines to learn statistical patterns based on large amounts of data. These algorithms and models are used with NDT and SHM systems to identify abnormal patterns or to predict a specific class based on knowledge-based data. ML algorithms are also utilized to extract features from input samples [28]. These algorithms are used in three broad approaches: supervised, unsupervised, and semi-supervised models. Supervised models are used to classify input observations to one of the labeled classes. A large size of data samples is used to train the models. Then, unseen data examples are used to test and validate these models. Examples of ML-supervised algorithms that are used with the SHM systems are decision trees, random forests, and support vector machines (SVMs). The k-means algorithm is a common model that is used to cluster the input samples into a set number of different groups. The state-of-the-art machine learning techniques are deep learning (DL) algorithms [27]. These algorithms are based on artificial neural networks. In the past decades, many efforts have been achieved in the field of computer vision. These efforts have been regularly improved to address issues with complicated visual patterns, including the detection of individuals, cars, and animals. A convolutional neural network (CNN) is one of the best and most common algorithms that is used with computer vision. Generally, it has an identical structure consisting of stacked convolutional layers followed by the contrasting normalization and maxpooling of one or more fully connected layers [29]. Many studies were conducted using CNN methods to address the issues of structural damage localization and identification [29,30]. The CNN methods have been successfully utilized in classifying images with very high accuracy. These methods can recognize complicated classifier physical boundaries and extract characteristics that can differentiate between various problem factors [28]. CNN models are artificial neural networks with one or more convolutional layers that are utilized for the processing, categorization, and segmentation of images [31]. CNN models have achieved ground-breaking accomplishments in a variety of feature recognition-related fields. The fact that CNN models minimize the number of artificial neural network (ANN) variables is their most beneficial feature [2]. CNN methods recently emerged as the most popular form of deep learning techniques because of their capacity to acquire knowledge directly from the initial signals in a sizable dataset [31]. Using complex filters and mappings of features, CNN models have been applied to pixel-level classification inside an image to locate and identify different interesting items [32]. Recent developments in the field of computer vision have increased the awareness of such NDT and SHM technologies as some of the greatest and most powerful techniques. Categorization, object localization, and pixel-level division are common recognition techniques for structural damages using ML. The collected data are classified as damaged or non-damaged using a proposed CNN based on the sliding window approach [33,34,35]. A new DL method combined with artificial neural networks was used to extract the features of measured guided waves during the fatigue test on composite plates. The laser technique was used to provide the dataset by scanning the test area after exciting it with the guided wave by installing sensors over the test plate. The results reflect the capability of the proposed system for identifying the plate damage [36]. A review study was implemented to present the progress in using ML methods for predicting the structural integrity and fracture of 3D-printed components [37]. Recent developments in sensor technology have increased the use of data-driven systems for SHM. Since advanced techniques and DL are linked, the requirement for sensors will decrease, bringing down the price of SHM and increasing the quality [38,39].

Four proposed supervised CNNs for damage classification were examined. These CNNs have different sizes of receptive fields. The results show that these methods can classify the datasets of cropped images from 3D pavement images with an accuracy of 94%. Also, the result shows a reverse relation between the training time and the size of the receptive field [40]. The pre-trained CNN has been recently used to enable crack-length estimation in metallic structures from the processing of acoustic emission (AE) signals without prior knowledge of AE history [41]. The applications of CNNs have been widely used for detecting and classifying various damages in composite structures. A new system was presented for identifying the location and size of impact events on a composite panel. This system includes a CNN-based metamodel with a network of passive sensors installed on the tested structure to receive the impact signals. The result demonstrates the capability of the system to detect impact events with an accuracy of 95% [42]. An interesting approach was proposed based on the CNN for differentiating between pristine and damage cases and predicting and classifying 12 different forms of delamination in composite laminate structures. The structural transient responses were transferred to spectrograms using STFT to be used as input data to the pre-trained CNN. The confusion matrix was obtained for evaluating the CNN, which came up with an accuracy of 90.1%. [43]. The damages in CFRP composite structures can be detected with data scarcity using the proposed transfer learning methodology to train an existing CNN. Structural vibration data were used to confirm the proposed method [44]. A new fully convolutional layer (FCN) model with semantic segmentation was presented for identifying the delamination shape, size, and location in composite structures. Numerical and experimental works were implemented on guided wave propagation and interaction with one delamination. For each damage case, one image was prepared by applying the RMS to the full-time wavefield. This RMS image was used as input data into the proposed FCN to be segmented into pristine and damaged parts to indicate the damage information. The results approved the capability of the FCN model for delamination identification compared with the traditional wavenumber filtering method [45]. An improved Global Convolutional Network (GCN) was adopted to characterize the CFRP plate delamination using a public dataset of wavefield images of guided wave propagation and interaction with damage. The input data can be one resulting image determined with the RMS technique or 3D wavefield animation. These two different data were obtained at three different resolutions and used as input data to different improved networks. The result approved the capability of the GCN to precisely identify the damage location and shape even with a low-resolution grid [46]. A new CNN-based semantic division method was developed in conjunction with the SLDV technique to identify structural delamination. The time sequence of wavefield images of Lamb wave propagation and interaction with delamination was used directly as input data to the novel DL. The results reveal the capability of this proposed model for identifying the mapping of damage [47]. The CNN can be used with acoustic steady-state excitation spatial spectroscopy for identifying the damage location, size, and shape based on predicting the plate thickness at each plate pixel. The results indicate the ability of the proposed CNN to precisely predict the plate thickness at a zone where the dispersion of Lamb waves is complex [48].

Automating the process of detecting and quantifying structural damages such as delamination is a necessary step to prevent catastrophic events and to perform the process of maintenance. Based on the literature review, there is limited research on using CNN methods for detecting the delamination depth across the composite plate thickness. The contribution of the present paper includes developing an automated system qualified for distinguishing between pristine and delamination structures and classifying three classes of delamination with various depths. This system involves a proposed CNN model based on the Lamb wave technique. The three datasets that are used independently as input data to the CNN model involved numerical and experimental wavefield images and experimental wavenumber spectrum images. The proposed CNN model results of the three different datasets were validated using the GoogLeNet CNN.

## 2. Test Specimen

**Figure 1:**
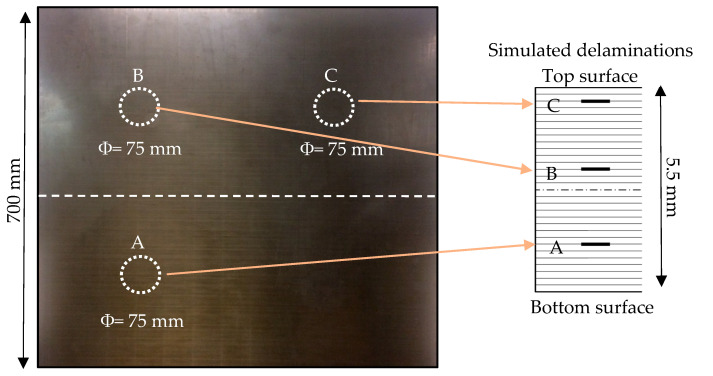
The illustration of the test plate (5.5 mm unidirectional [0]_30_ CFRP composite plate) shows three simulated delaminations inserted at different depths.

In the present work, a unidirectional CFRP composite plate with a stacking sequence of [0]_30_ and dimensions of 700 mm × 700 mm × 5.5 mm was utilized for the numerical and experimental investigations. The three delaminations were created in this test composite plate by putting Teflon material pieces at different depths throughout the thickness of the plate through the manufacturing process of the test specimen, as shown in Figure 1. The delamination C was simulated near the top surface by inserting Teflon material between layers 2 and 3. Delamination B was created between layers 12 and 13 and delamination A was created between layers 23 and 24. The details of preparing the test specimen are explained in the recent work [12]. Table 1 shows the mechanical properties of the test unidirectional CFRP composite plate.

## 3. Studying Delamination Detection Using Guided Waves

Numerous accomplishments have been achieved in detecting and assessing the delaminations in composite structures utilizing measured time–space signals obtained from a scanning laser Doppler vibrometer (SLDV). These wavefield data of the scanning area illustrate guided waves propagating in the test composite plate as well as guided waves interacting with the three different delaminations. The wavefield information can be examined for additional analysis using various techniques to measure structural damages such as wavefield energy map, and imaging-based local wavenumber analysis [49,50]. This section includes numerical and experimental studies to examine the propagation of Lamb wave modes in the delamination composite plate and to observe the interaction of these wave modes with delamination. The computational work involved using the finite element (FE) simulation to build a 3D simulation model. The experimental study was conducted to validate the numerical result using SLDV. The numerical and experimental obtained wavefield data were investigated to find the delamination and notice how the depth affected the scattered and trapped waves formed over the delamination zones. In addition to this study, the main objective of this section was to prepare three datasets of numerical and experimental wavefield images and experimental wavenumber spectrum images that were used independently as input datasets to the proposed CNN model and the GoogLeNet CNN to detect and classify the delaminations as a function of their depth.

### 3.1. Multi-Physics Finite Element Simulation

**Figure 2:**
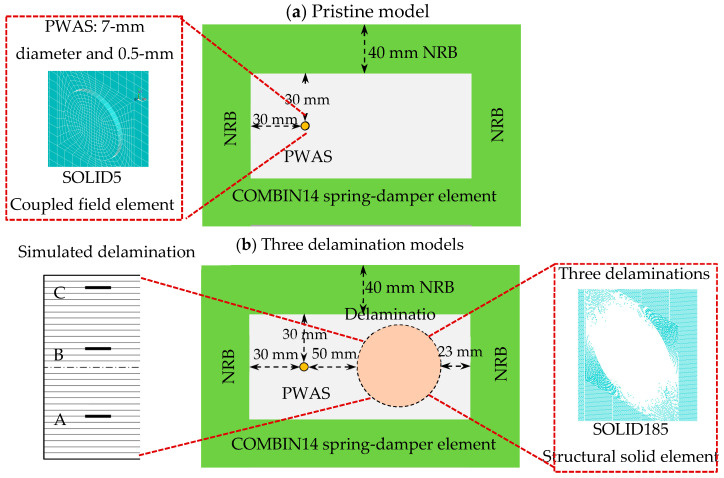
Finite element models of the unidirectional CFRP composite plates; (**a**) pristine model; (**b**) three delamination models.

Four FE 3D models were created to numerically examine the propagation of the guided wave in a unidirectional composite plate and to study the interaction of this wave with three different cases of delamination. These models are one pristine model and three delaminations, A, B, and C. Figure 2a illustrates the FE simulations of Lamb wave propagation in the pristine CFRP composite plate. Figure 2b illustrates the FE simulations of Lamb wave propagation and interaction with three cases of delamination. The delamination model (Figure 2b) was modeled by detaching the nodes [12]. The delamination is produced between two distinct layers by identifying it as two distinct planes with the same coordinates but no link. A 75 mm delamination depth for the three simulated cases was selected as the same as the test specimen (delamination C, between plies 2 and 3; delamination B, between plies 12 and 13; and delamination A, between plies 23 and 24). The non-reflective boundaries (NRBs) are created around the 3D FE-simulated models to avoid border reflections and to get small-size models [51]. The numerical time–space wavefield data were measured under PWAS excitation. The signal of excitation used in this simulation is a three-count Hanning window-modulated tone burst with a center frequency of 120 kHz. The ANSYS element SOLID5 was used to model PWAS. The composite plates have meshed using SOLID185. The largest element size that can be utilized for ensuring mesh convergent is adopted using Equation (1) [52].

(1) λminle≥20

**Figure 3:**
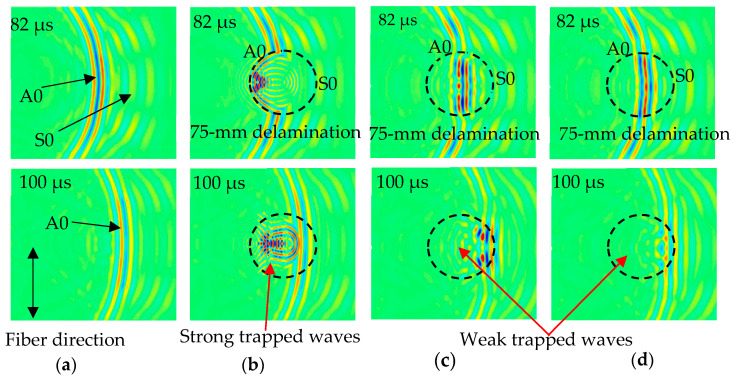
Numerical wavefield images of four scanning tested areas measured at times 82 μs and 100 μs; (**a**) pristine; (**b**) delamination C; (**c**) delamination B; and (**d**) delamination A. The frequency of guided waves is 120 kHz.

λ_min_ represents the smallest wavelength and *l_e_* represents the adopted element size. In this simulation, the adopted element length is 0.5 mm in the surface direction and 0.2 mm in the thickness direction. This is determined based on the wavelength of the A0 mode, which is around 10 mm in this study. The NRB was constructed using the spring-damper components from the COMBIN14. Figure 3 illustrates the numerical wavefield at different time steps of the Lamb wave propagation in the pristine CFRP composite plate and propagation and interaction with three cases of delamination in CFRP composite plates. Based on the wavefield images at time 82 μs, it can be concluded that the damage depth has a considerable impact on guided wave interactions with delamination. Due to its small energy (low amplitude), the S0 mode has little interaction with delamination. The significant amplitude of the A0 mode creates a powerful wave–damage interaction. The A0 Lamb mode shows a substantial interaction with delamination C due to its nearness to the top surface, as illustrated in Figure 3b. However, a weak interaction can be observed for cases of delamination B and A, as illustrated in Figure 3c,d, because they are inserted far from the top surfaces. However, delamination B has a stronger interaction with the A0 Lamb wave mode than delamination A. Because delamination C is located near the scanning surface (top surface), it contains more trapped wave energy over the delamination zone than the other two cases (see the wavefield images at time 100 μs). In brief, reductions in trapped wave density and energy can be observed as the delamination depth increases. It is possible to determine the size, position, and shape of the delamination under various investigation methods using these trapped waves.

### 3.2. Experimental Validation Setup

**Figure 4:**
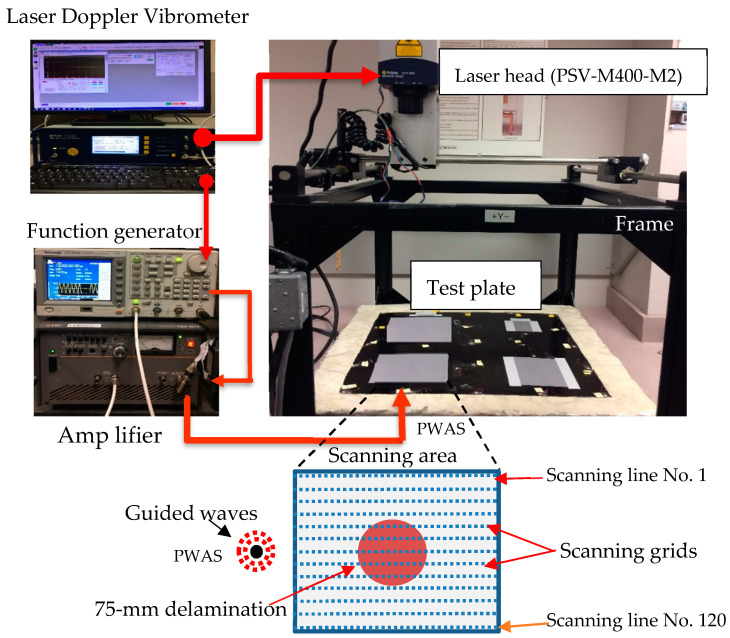
Setting up the experiment with the SLDV technique to investigate the Lamb waves–delamination interaction in the unidirectional CFRP composite plate.

**Figure 5:**
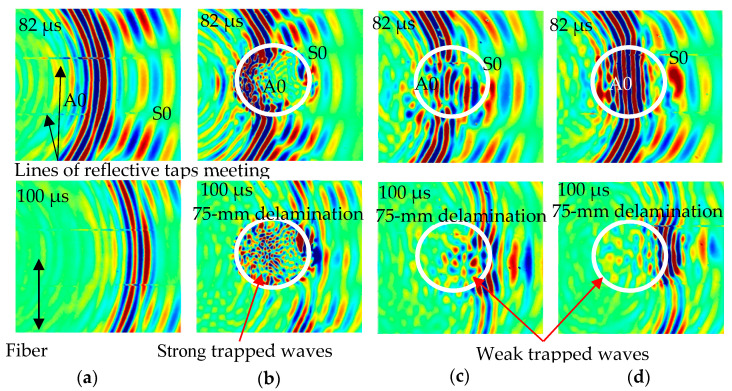
The experimental time–space wavefield images at times 82 μs and 100 μs: (**a**) pristine case, (**b**) delamination C, (**c**) delamination B, and (**d**) delamination A. The frequency of guided waves is 120 kHz.

Validation experiments were conducted to assess the computational results from the previous part. The combination of the PWAS transducer with SLDV can be used to investigate the interaction of guided waves with delaminations. Figure 4 illustrates the setup of the conducted experiment. Four scanning areas (one pristine and three delamination areas) were prepared with appropriate dimensions and covered by reflecting tape to enhance the quality of the scanning area signals. (Note to the reader: The use of reflective tape is standard practice as recommended by the SLDV manufacturer. Unfortunately, it leads to measurement artifacts in the form of lines delineating the tape boundary. This is the reason for some horizontal lines that the reader may see in the SLDV images shown in Figure 5). Four PWAS transducers (APC 850, 7 mm diameter, and 0.2 mm thick) were installed at an appropriate distance from each scanning area. Using the same numerical simulation, a function generator produces the three-count tone burst signal, which is amplified by a power amplifier and has a center frequency of 120 kHz. The amplifier is connected to each of these four PWAS transducers to trigger Lamb waves in the test scanning area. The laser’s head (PSV-M400-M2) was used to acquire the time–space wavefield of the 130 mm × 120 mm area for each scanned area with a 1 mm spatial resolution. Each of the four scanning areas is divided into 120 scanning lines in the direction of propagating guided waves. Each line has 130 spatial points. The laser’s head generates a light source orthogonal to the scanning region to measure the out-of-plane motion of each scanning point. The quality of the measured signal at each scanning point was improved by averaging it 20 times. For extracting the test plate delamination information (location, shape, and size), the wavefield data obtained for each of the scanning areas can be assessed.

### 3.3. Experimental Validations Results

**Figure 6:**
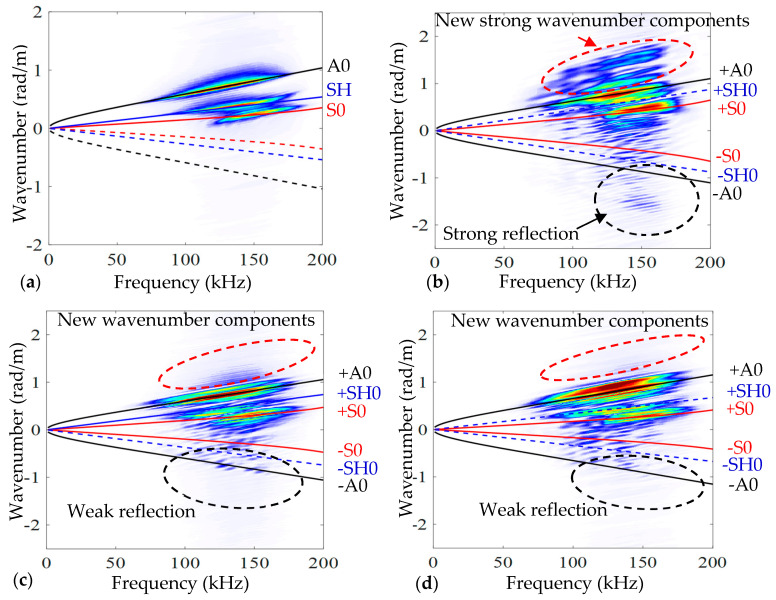
The wavenumber-frequency spectra of lines scan across the centers of four scanning areas: (**a**) pristine; (**b**) delamination C; (**c**) delamination B; and (**d**) delamination A. The scanning area is divided into 120 scanning lines in the direction of propagating waves. The wavenumber–frequency spectrum was obtained for each scanning line.

The SLDV can be utilized for collecting time–space data for each point in the scanning region. These scanning points have time domain signals (measured guided wave signals) which are useful for obtaining time–space wavefield images of the investigated area, as illustrated in Figure 5. Two Lamb wave modes are apparent in all four cases and these guided waves are moving across the test plate in a transverse direction. The A0 mode is slightly slower and stronger than the S0 mode, as illustrated by the wavefield images at time 82 μs. When comparing delamination C to each of the two cases (delamination B and delamination A), the interaction with A0 is considerably more apparent, which is consistent with FE data. Based on the wavefield images at time 100 μs, trapped waves can potentially be seen in the three delamination zones following the interaction of the A0 mode with the delamination. Compared with the other two cases, because delamination C is located near the scanning surface, it shows strong, concentrated trapped waves over the delamination zone. The position, dimensions, and shape of the delamination can be determined using these trapped waves. Based on the above experiment wavefield images (Figure 5), when the delamination depth becomes greater (cases B and A), the generated trapped waves over delamination zones become weaker and less intense, as indicated quantitatively in the numerical results (Figure 3). According to the computational and experimental data, the potential energy of trapped waves across the delamination zone can be affected by the depth of delamination. The SLDV scanning area has a group of scanning lines in the direction of propagating waves. Each line includes a group of scanning point data (spatial signals) that can be utilized to obtain a frequency–wavenumber spectrum by applying a two-dimensional fast Fourier transform technique. The investigated delamination can be found using the frequency–wavenumber spectrum. In this work, each of the four scanning areas is divided into 120 scanning lines in the direction of propagating guided waves. The frequency–wavenumber spectrum was obtained for each of the 120 scanning lines. The wavenumber spectra obtained for the scanning lines across the centers of the pristine and three damaged scanning areas are displayed in Figure 6. The wavenumber spectra included in Figure 6 show an excellent fit between the experimental positive wavenumber spectra of the Lamb wave modes A0, SH0, and S0, and the analytical A0, SH0, and S0 positive wavenumber curves.

Because of its closeness to the scanning surface, the delamination C spectrum contains new, significant wavenumber components on both the positive (forward propagation) and negative (reverse propagation) sides, as shown in Figure 6b. Based on Figure 6c,d, the spectra of delaminations B and A observe new weak positive and negative wavenumber components because delaminations B and A are inserted away from the top surface and have a weak interaction with guided waves. However, delamination B has a somewhat greater number of wavenumber components than delamination A.

## 4. Datasets for CNN Models

All data-driven approaches in SHM require data as a fundamental component. With the emergence of DL-based techniques that are capable of handling and analyzing massive volumes of data, the importance of reliable data for SHM systems has become clear [53].

**Figure 7:**
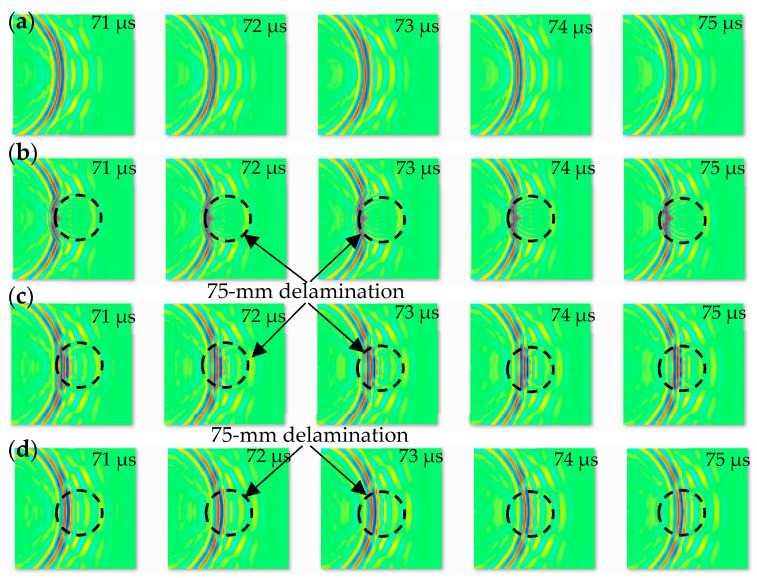
1004 numerical time series wavefield images (224 × 224 pixels) were prepared to be used as input datasets to the proposed CNN model and GoogLeNet CNN. These images are classified into four classes (251 images for each class): (**a**) pristine; (**b**) delamination C; (**c**) delamination B; and (**d**) delamination A.

In this work, we have three different datasets consisting of images prepared from numerical and experimental works. From the numerical work, the wavefield images represent time series images of guided waves propagating and interacting with delamination. We prepared a MATLAB code to customize and increase the resolution of the images, crop, and resize the images to be 224 by 224 pixels. The 1004 numerical images were prepared with 224 × 224 pixels before being used as input data to the designed CNN and the existing GoogLeNet CNN. These images were classified into four classes with 251 images for each class. The first class has wavefield images of the pristine case. The second, third, and fourth classes have wavefield images of the delamination C, B, and A cases, respectively, as demonstrated in Figure 7. For the three delamination classes, the scattered, reflected, and trapped waves represent the variations that exist between the images in a set of input data. The characteristics of these waves are dependent on the delamination depth. Based on this, each class of wavefield images has different features. All the images of the three delamination classes show either S0 mode (faster mode) interaction with delamination or A0 mode (slower mode) interaction with delamination. Two steps are adopted to obtain images with the wavefield–delamination interaction. In the first step, the PWAS transducer was installed at an appropriate distance from the scanning area to make sure that the S0 mode could reach all the spatial points of the scanning area and interact with delamination. In the second step, the first wavefield image of each class is obtained at the time step when the S0 mode reaches all the scanning points.

**Figure 8:**
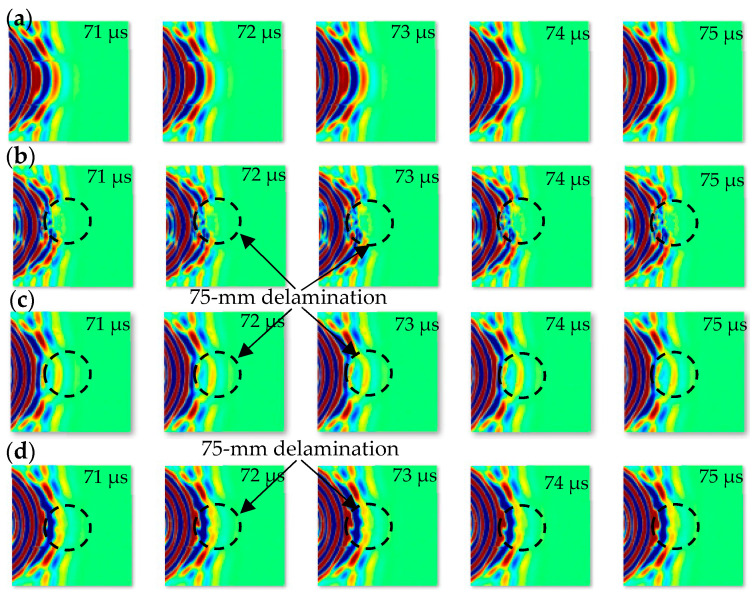
2804 experimental time series wavefield images (224 × 224 pixels) were used as input datasets to the proposed CNN model and GoogLeNet CNN. These images are classified into four classes (701 images for each class): (**a**) pristine; (**b**) delamination C; (**c**) delamination B; and (**d**) delamination A.

**Figure 9:**
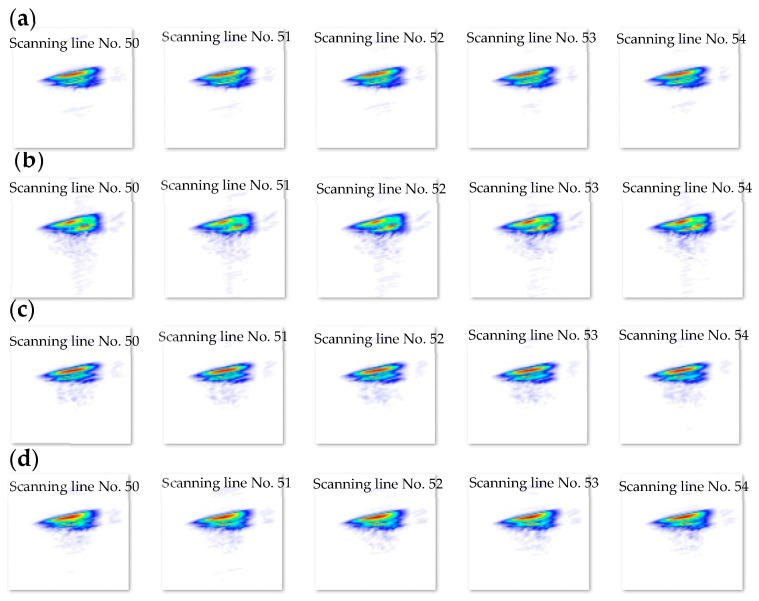
480 experimental wavenumber spectrum images (224 × 224 pixels) used as input data to the proposed CNN model and the GoogLeNet CNN. These images are classified into four classes (120 images for each class): (**a**) pristine; (**b**) delamination C; (**c**) delamination B; and (**d**) delamination A.

For the experimental work implemented by SLDV, there are two datasets which are wavefield images and wavenumber spectrum images. The time–space wavefield dataset includes 2804 time series images distributed equally in four classes (each class has 701 images), as shown in Figure 8. We can observe that a higher number of experimental wavefield images were used compared to the numerical wavefield images. This is because the time step (time interval) of the numerical guided wave signal is larger than the time step of the experimental work with the same time length of the guided wave signal. The numerical work, with the same experimental time steps value, needs a computer with high specifications and high processing time to implement the simulation. Based on this reason, we adopted an appropriate time interval for numerical simulation that makes the number of numerical wavefield images less than the number of experimental wavefield images. The wavenumber spectrum images dataset that can be obtained by processing the time–space wavefield of each scanning line includes 480 images distributed equally in four classes (each class has 120 images), as shown in Figure 9. Each wavenumber spectrum reveals the information of full-time measured signals of spatial line scan points. These images were modified to fit the 224 × 224 pixels’ standard and classified separately into four classes before being provided into the input layer of the proposed CNN model and GoogLeNet CNN.

## 5. Delamination Detection and Classification Using CNN

**Figure 10:**
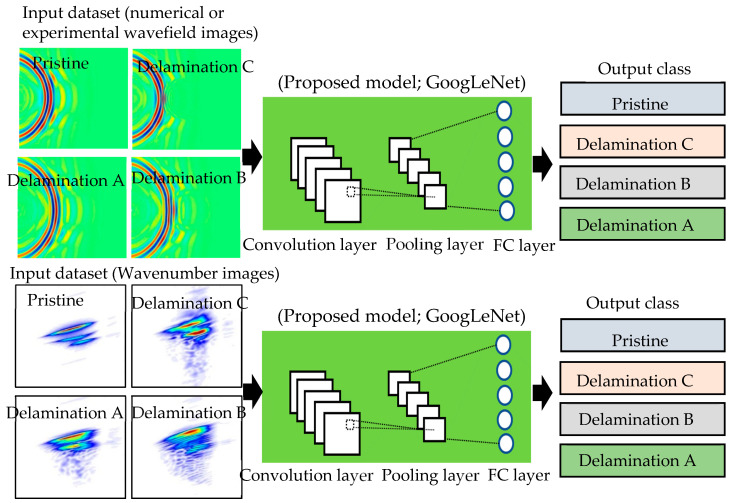
Schematic of using the proposed CNN model and adopted GoogLeNet CNN for detecting delaminations and predicting their depths.

As illustrated in Section 4, the three datasets (numerical wavefield images, experimental wavefield images, and experimental wavenumber spectrum images) were used independently as input data for the training, validation, and testing of the proposed CNN model and GoogLeNet CNN. The proposed CNN model classified each of these three datasets into four classes (one pristine class and three different delamination classes). The delamination classes are classified based on the delamination depth. The results of the proposed CNN model were validated using the GoogLeNet CNN, as shown in Figure 10.

### 5.1. Proposed CNN Model

**Figure 11:**
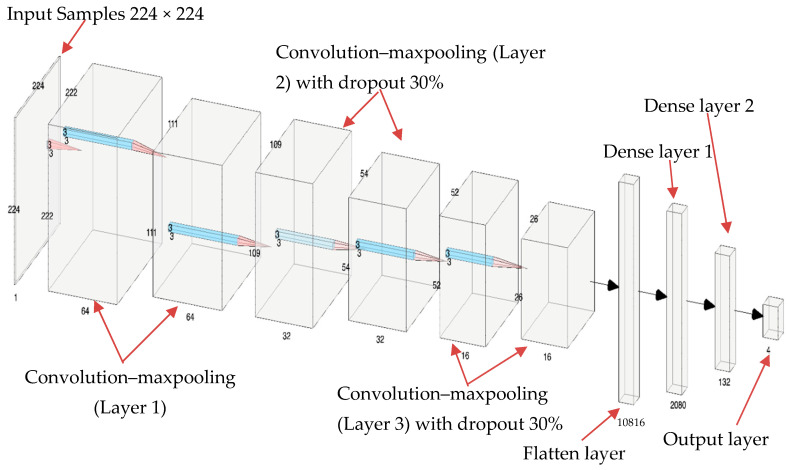
The architecture of the proposed CNN model.

This part contains the details of designing the proposed CNN-based delamination identification system. The proposed CNN-based system consists of one input layer, three CNN–maxpooling layers, one flattened layer, two dense layers, and one output layer, as shown in Figure 11. The input layer reads a sample of 224 × 224 pixels in dimensions. Then, a convolutional layer performs convolution processes using 64 filters with 3 × 3 dimensions with a stride of size 1. The ReLU activation function is used with the convolutional operations to evaluate the feature signal to a weak or strong signal. These processes produce 64 feature maps, each with a size of 222 × 222 pixels, which present the features of the input image–plate sample. The dimensions are reduced to 222 × 222 pixels because the used filter sizes are 3 × 3 and the dimensions of the input sample are 224 × 224 pixels, which is not a multiple of three. In addition to that, we use no padding with zeros in the convolutional processes to fit the dimensions. Next, maxpooling operations are applied to extract the main features using a window of size 2 × 2. The maxpooling layer reduces the size of the feature map size to half, resulting in a size of 111 × 111. Then, two more convolution–maxpooling layers are added to focus on the most important features and reduce the feature map size. The only difference is the number of filters in the second and third convolution layers, which are set to 32 and 16, respectively. The results of the three convolution–maxpooling layers are 16 feature maps with sizes of 26 × 26. Next, these feature maps are flattened to a big one-dimensional array with a size of 10,816. The flattened layer is fed to two dense layers with sizes of 32 and 16 nodes, respectively. Finally, the model ends with an output layer of four nodes to classify the plate sample into one of four classes (delamination A, delamination B, delamination C, or pristine). To evaluate the input sample to one of multiple classes, the Softmax activation function is used in the output layer. To generalize the models’ behavior and avoid overfitting, two dropout layers were added next to the second and third convolution–maxpooling layers. The dropout rate is set to 30 percent, which means 30 percent of weights will be dropped during training iterations.

The proposed CNN model has specific advantages compared to existing models, which can be explained in the following points:

1. The proposed CNN model is less complex compared to the pre-trained model in terms of the number of trainable parameters.
2. The structure of the proposed model is clear, and the dimensions of the input and output sample in each layer are obvious and easy to track.
3. A less complex model (relatively small number of trainable parameters) means it is easy to optimize the model behavior in terms of updating the hyperparameters. In turn, this leads to reducing the consumption of power resources and training time.

Table 2 below presents the total number of trainable parameters and the trainable parameters for each layer of the proposed CNN model.

Generally, two main factors affect the detection performance of the proposed models in terms of model design. The first factor deals with specifying the number of features that are used to identify the delamination. The number of feature maps is determined by setting the structure of the CNN layers in the model, which means specifying how each layer receives a form of input sample, processes it, and generates an output shape. This process will continue until the fully connected layer where the identifying features are ready to be used in the output layer. The second factor is the number of trainable parameters. The trainable parameters reflect model behavior, and its number depends on the design of each layer. Trainable parameters represent the weights that are used in the model to recognize the delamination and the delamination type by extracting the main features. The number of trainable parameters is important to enhance the model capacity. However, increasing the number of trainable parameters could lead to overfitting issues and reduce model generality. Moreover, increasing the number of trainable parameters will increase the model complexity in terms of resource consumption and processing time. Therefore, we proposed a model with a smaller number of trainable parameters and high performance compared to pre-trained models.

### 5.2. Evaluation Metrics

To test the behavior of the proposed CNN model, and the adopted GoogLeNet CNN, four metrics are used: accuracy, precision, recall, and F1 score. This section presents a theoretical analysis of the evaluation metrics. To evaluate the overall behavior of the models, an accuracy metric is used. Accuracy is estimated by dividing the number of correct predictions by the entire number of predictions (the summation of correct and false predictions), as shown in Equation (2) [54]. The correct predictions represent the summation of true positive (TP) and true negative (TN) samples. The false predictions represent the summation of negative samples falsely predicted as positive (FP) and positive samples with a negative (FN) prediction.

(2) $$\mathrm{Accuracy}=\frac{\mathrm{TP}+\mathrm{TN}}{\mathrm{TP}+\mathrm{TN}+\mathrm{FP}+\mathrm{FN}}$$

The precision metric is used to see the percentage of positive samples that are predicted correctly, i.e., the true positives (TPs), against the total number of samples that are predicted as positive (see Equation (3)). Moreover, the recall metric is applied to measure the rate of the number of true positive (TP) samples that predicted correctly to the entire number of correct predictions (see Equation (4)). Finally, the F1 score is used to present the balance between precision and recall. The main concept of the F1 score is to weight the two ratios and show their average, as illustrated in Equation (5).

(3) $$\mathrm{Precision}=\frac{\mathrm{TP}}{\mathrm{TP}+\mathrm{FP}}$$

(4) $$\mathrm{Recall}=\frac{\mathrm{TP}}{\mathrm{TP}+\mathrm{FN}}$$

(5) $$F1\;\mathrm{score}=\frac{2\times \mathrm{Precision}\times \mathrm{Recall}}{\mathrm{Precision}+\mathrm{Recall}}$$

### 5.3. Results of the Proposed CNN Model

This section presents our findings of the CNN-based proposed model, as shown in Figure 11. The proposed CNN model is trained with three different datasets (numerical wavefield images, experimental wavefield images, and wavenumber spectrum images) that are randomly divided into three subsets (70% of data to be used for training, 10% of data to be used for validation, 20% of data to be used for testing). For each dataset, we illustrate learning and loss curves, and a confusion matrix. A confusion matrix is a list of values used to describe how well a classification system performs. It shows and sums up a categorization method’s accuracy. The confusion matrix has a horizontal axis that represents the predicted classes (column by column). The vertical axis represents the true classes (row by row). The correct classification of samples of each class is placed on the diagonal of the confusion matrix. The misclassification samples may occur and are placed off-diagonally in the confusion matrix. Moreover, we provide a model-behavior analysis for each of the used datasets using four evaluation metrics (accuracy, precision, recall, and F1 Score).

#### 5.3.1. Results and Discussion of Numerical Wavefield Dataset

**Figure 12:**
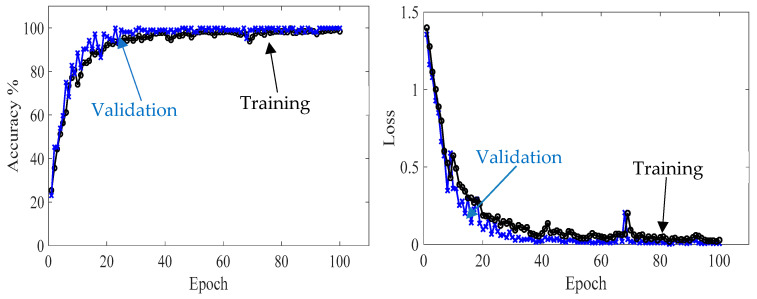
Training progress curves show the accuracy and loss varying with iteration (training process). The numerical wavefield dataset is used as the input dataset to the proposed CNN model.

**Figure 13:**
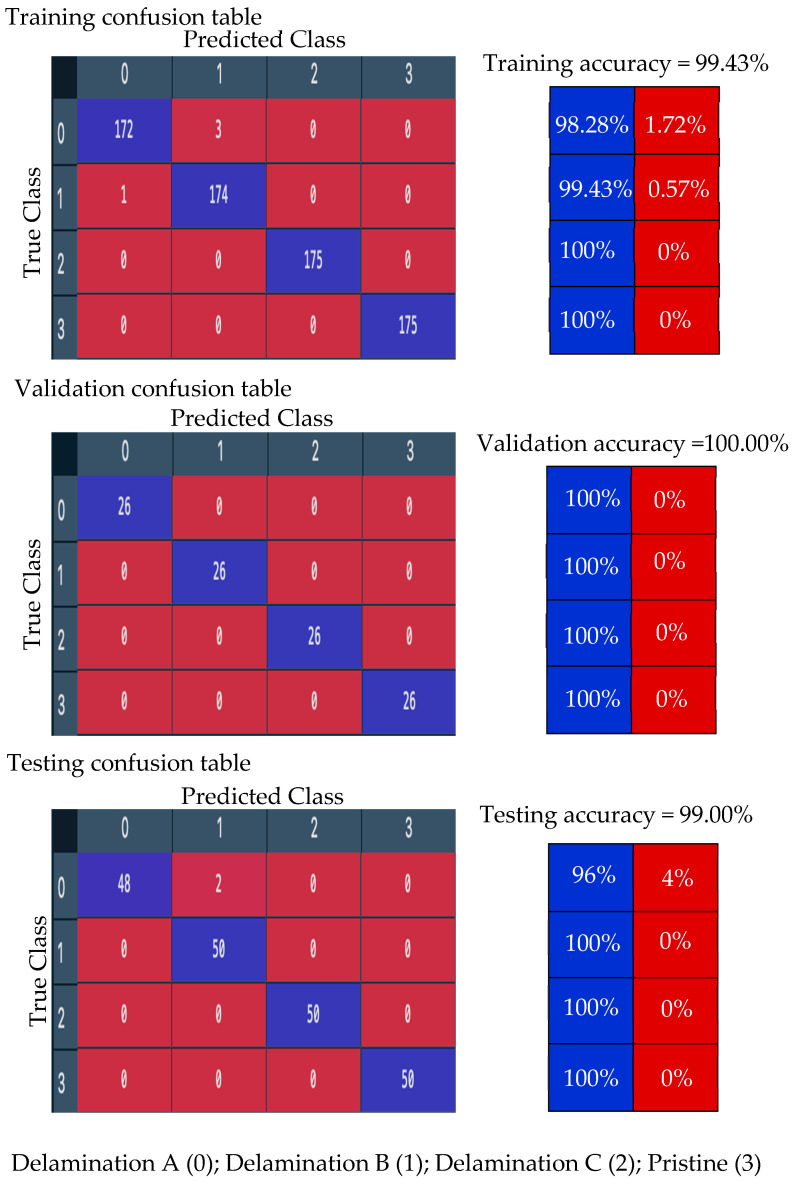
Measured confusion matrices for training, validation, and testing the proposed CNN model using input numerical wavefield dataset.

First, the proposed CNN model is trained with 70% of the numerical wavefield dataset (700 samples of four classes, 175 samples for each class). The training process shows smooth learning curves for both training and validation data points, as shown in Figure 12. Four curves are presented for the accuracy and loss of training subset. The number of iterations is set to 100 epochs. The model is converging at epoch 100 with a training accuracy of 99.43%, and a validation accuracy of 100%; see the confusion metrics in Figure 13.

The model is tested with 200 samples of four classes distributed evenly, with 50 samples for each class (20% of the numerical wavefield dataset). Table 3 shows the evaluation metrics that describe the model behavior of the testing subset. The model identification behavior of each class is compared to other classes in terms of precision, recall, and F1 score. All these metrics measure the accuracy of the model from different perspectives. The precision of the first class, delamination A, is 1, which means there are no other classes predicted as delamination A. However, the situation is different with delamination B, where precision equals 0.96. This indicates that other classes are falsely predicted as delamination B. From the confusion matrix of the testing subset, shown in Figure 13, we can see that two samples from delamination A are predicted as delamination B.

The recall value of the first class, delamination A, is 0.96. This means some samples of delamination A are predicted with different labels. Two samples of delamination A are predicted as delamination B; see the confusion matrix of the testing subset in Figure 13. By noticing the precision and recall values, we see that the model identifies class delamination C and the pristine class perfectly, as shown in Table 3 and Figure 13. The F1 score presents the balance behavior between precision and recall. It is the harmonic mean of precision and recall. The F1 score is sensitive to the low value of either precision or recall. If the model showed a weak behavior regarding any class, the F1 score would present a low score regardless of the number of samples of that class. From Table 3, we can see that the weighted average of the F1 score is 0.99, which means the proposed CNN model identifies most of the observations of four classes correctly.

#### 5.3.2. Results and Discussion of Experimental Wavefield Dataset

**Figure 14:**
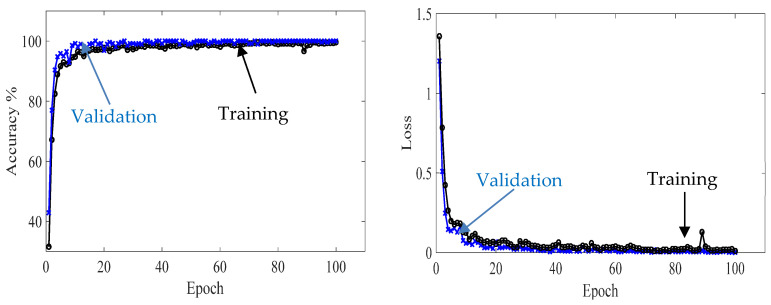
Training progress curves show the accuracy and loss varying with iteration (training process). The experimental wavefield dataset is used as the input dataset to the proposed CNN model.

**Figure 15:**
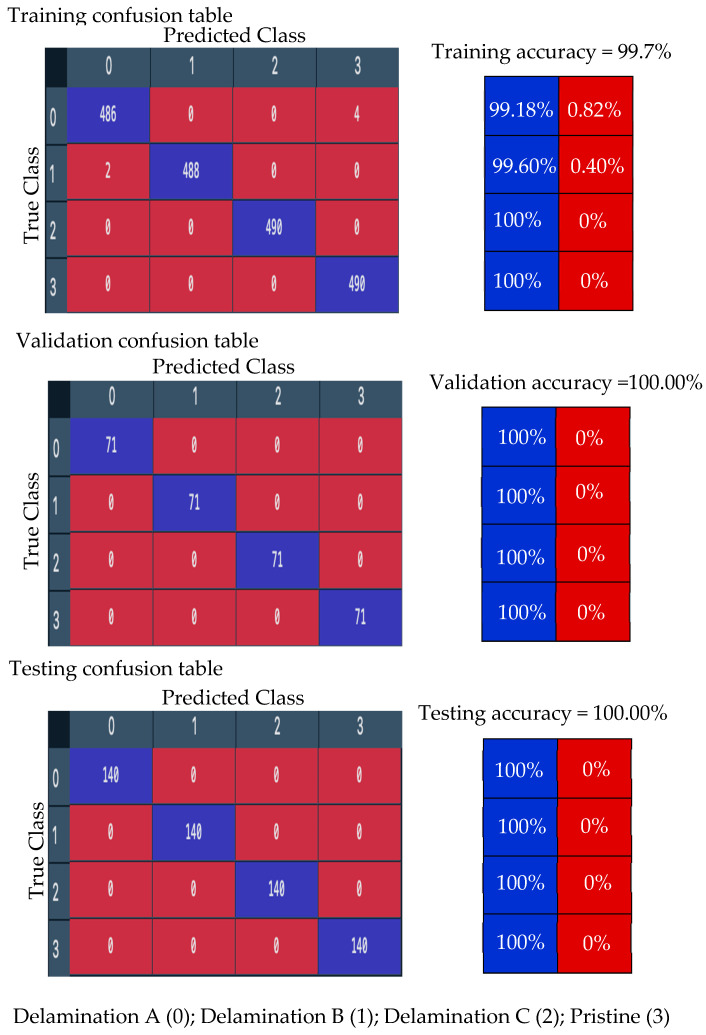
Measured confusion matrices for training, validation, and testing the proposed CNN model using input experimental wavefield dataset.

Second, the proposed CNN model is trained with 70% of the experimental wavefield dataset (1960 samples of four classes, 490 samples for each class). The model behavior through training and validation is presented in learning curves, as shown in Figure 14. The model is converging at epoch 100 with a training accuracy of 99.7%, and a validation accuracy of 100%; see the confusion metrics in Figure 15. The proposed CNN model is tested with 560 samples of four classes distributed evenly, resulting in 140 samples for each class (20% of the experimental wavefield dataset). As illustrated in the confusion matrix of Figure 15, the model evaluates all of the tested image samples correctly. Also, Table 4 shows three evaluation metrics that describe the model behavior. All three metrics, precision, recall, and F1 score, present high scores with a value of 1.0. In short, the model classifies all samples accurately.

#### 5.3.3. Results and Discussion of Experimental Wavenumber Spectrum Dataset

**Figure 16:**
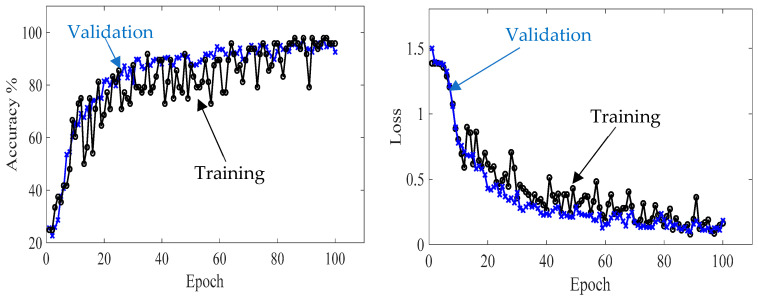
Training progress curves show the accuracy and loss varying with iteration (training process). The experimental wavenumber spectrum dataset was used as the input dataset to the proposed CNN model.

**Figure 17:**
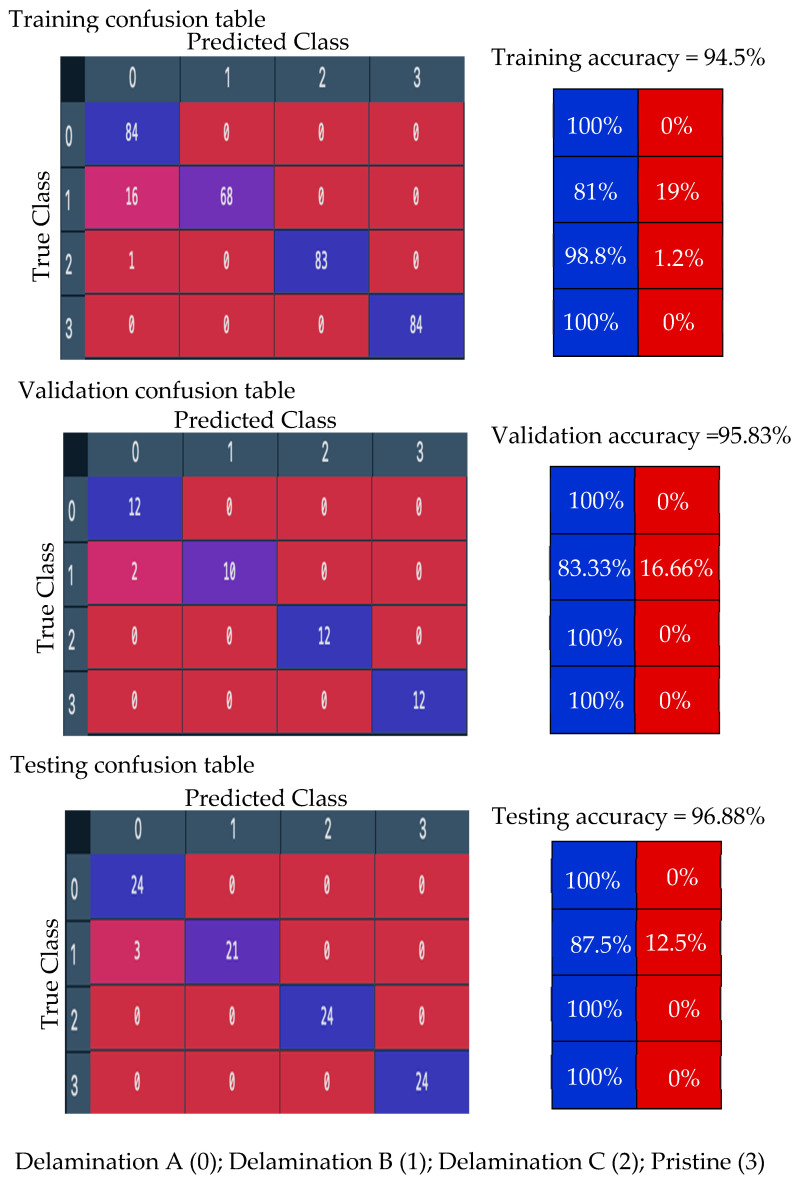
Confusion matrices for training, validation, and testing proposed CNN model using the experimental wavenumber spectrum dataset as input.

Third, the proposed model is trained with 70% of the experimental wavenumber spectrum dataset (336 samples of four classes, 84 samples for each class). The model is converging at epoch 100 with a training accuracy of 94.5%, and a validation accuracy of 95.83%; see the learning curves and confusion metrics in Figure 16 and Figure 17, respectively. The model shows a better performance with the testing dataset; see the confusion metrics in Figure 17. The recall value of the second class, delamination B, is 0.875. This means some samples of delamination B are predicted with different labels. Three samples of delamination B are predicted as delamination A; see the confusion matrix of the testing subset, Figure 17. Table 5 shows three evaluation metrics that describe the model behavior. The input image-based wavenumber spectrum data samples present the frequency domain of the raw signals, where each pixel reflects the frequency of a wavenumber value. The CNN-based model will process these pixels by applying different sizes of filters to extract different band–frequency features. These features are used to classify the input data sample to classes A, B, C, or the pristine class. However, the result is not promising because part of the spatial information in the original signals such as the wave propagation direction could be lost in the transformation from the raw signals into two-dimensional image-based samples. Another factor that leads to low performance in identifying delamination classes is the lack of a dataset. A big spectrum-based dataset is recommended to train the proposed model. All three metrics, precision, recall, and F1 score, present high scores with an average value of 0.97. In short, the model classifies most of the samples accurately. Despite the fact that the accuracy is not much different from the accuracy of the experimental wavefield image dataset, it is recommended to use a wavenumber spectrum dataset as an input dataset for several reasons. First, the wavenumber spectrum represents the frequency–wavenumber domain of the full-time measured signals of line scanning points. It has significant information that is important for various tasks such as CNN classification. Second, the wavenumber spectrum helps the CNN to significantly learn hierarchical features. Third, the visual frequency domain of the wavenumber spectrum can offer a good opportunity for the CNN to understand the main features for classification purposes. Fourth, the wavenumber spectrum data samples are slightly affected by experimental noise compared to the image-based wavefield data samples. For this, the wavenumber spectrum dataset is preferred to train the deep learning models since they present robust feature representation.

### 5.4. GoogLeNet CNN

The presented result of the proposed CNN model can be validated using the pre-trained GoogLeNet CNN [55,56]. The structure of the GoogLeNet model and the number of its trainable parameters can be obtained by using the summary method that comes with the Keras library [57]. The MATLAB deep learning toolbox was used to set up the GoogLeNet CNN. It was utilized to classify four classes of image datasets (pristine, delamination C, delamination B, and delamination A). GoogLeNet, which has 144 layers and 170 connections, is useful for computer vision applications like object recognition and image categorization. It was designed to have weighted Gabor filters of varying sizes in the inception sparse design, which enables it to be deeper as well as wider without needing more processing resources. This CNN needs images with a PNG extension and a size of 224 × 224 pixels as input data. To create image datastores and augmented image datastores (augment training image data with randomized preprocessing operations to help prevent the network from overfitting) for the four classes test, a MATLAB code was developed to randomly split the data into three groups (70% of data to be used for training the CNN, 10% of data to be used for validation steps during the CNN training, and 20% of data to be used for testing how good the trained CNN is). Next, the training and validation data were loaded into the deep network designer to train and validate the GoogLeNet CNN. A set of training options were selected for training the selected CNN. The stochastic gradient descent with momentum was chosen as a solver and the initial learning rate was set to a value of 0.0001. The validation frequency was set to a value of 5, the maximum epoch number was set to a value of 50, and the mini-batch size for the training was set to a value of 128. The training process will take some time depending on the size of the training data, the number of epochs, and the performance of the computer that is used. The training process can be monitored by plotting various metrics such as training and validation accuracy and loss metrics. After the training is finished, the trained GoogLeNet CNN can be saved and tested to verify its classification prediction. The test dataset is used for verifying the trained GoogLeNet CNN. A MATLAB code was developed to calculate the confusion matrix and display the prediction accuracy of the trained GoogLeNet CNN.

#### 5.4.1. Results and Discussion of Numerical Wavefield Dataset

**Figure 18:**
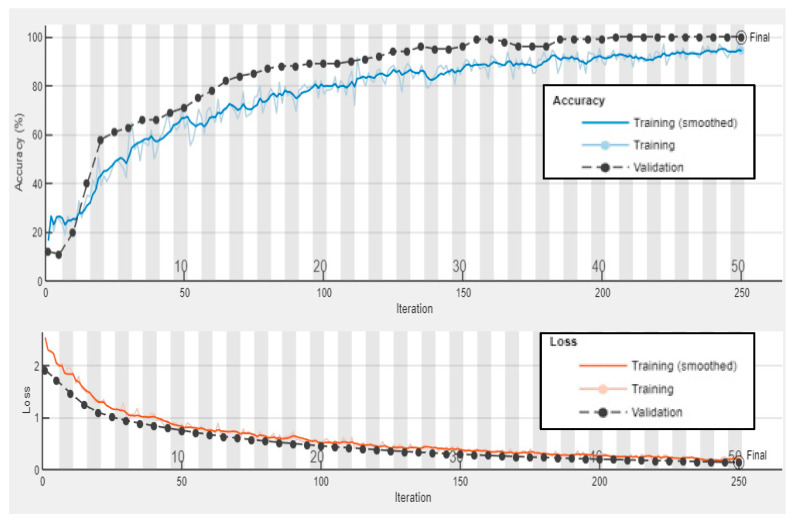
Training progress curves show the accuracy and loss varying with iteration (training process). The numerical wavefield dataset was used as an input dataset to the GoogLeNet CNN.

**Figure 19:**
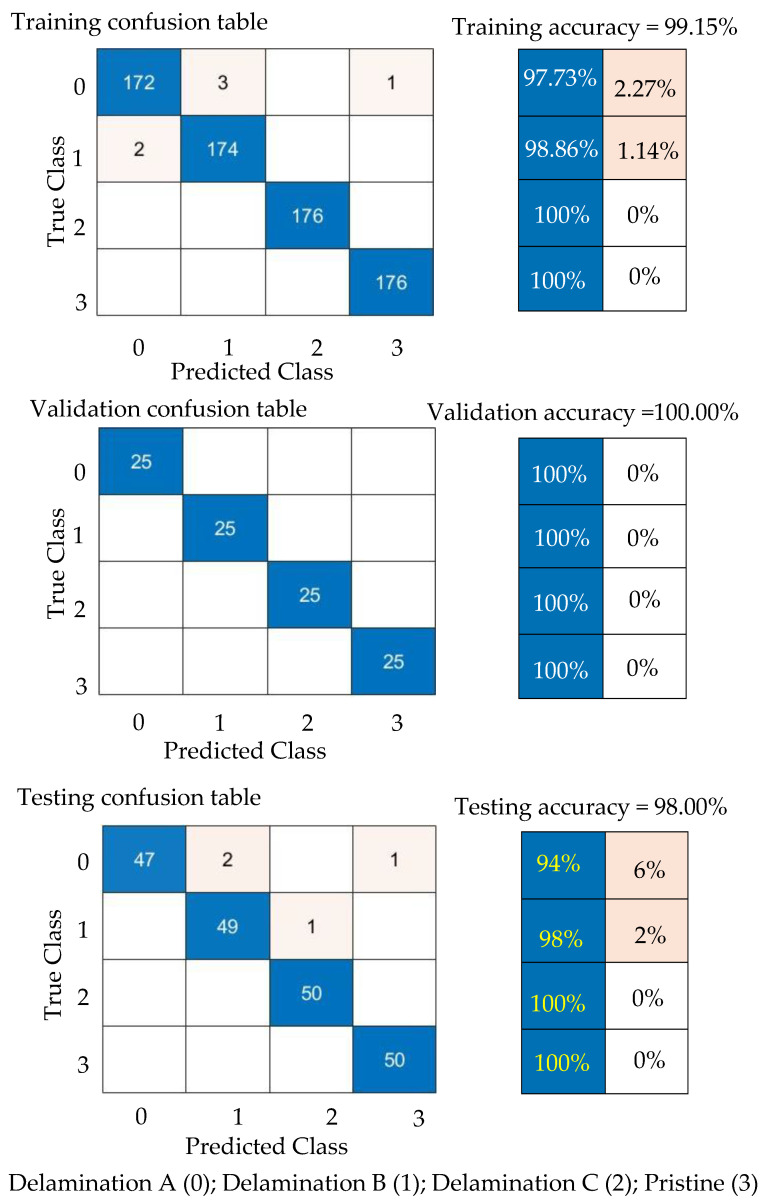
Measured confusion matrices for training, validation, and testing GoogLeNet CNN using the numerical wavefield dataset as input.

First, the GoogLeNet CNN is trained with 70% of the numerical wavefield dataset (704 samples of four classes, 176 samples for each class). Figure 18 shows the training progress of the GoogLeNet CNN. The observed typical accuracy and loss vary with the iteration, showing smooth learning curves. The trained GoogLeNet is converging at epoch 50 with a training accuracy of 99.15%, and a validation accuracy of 100%; see the confusion metrics in Figure 19, which is based on the classification report of the test dataset (Table 6). The precision of the first class, delamination A, is 1.0, which means there are no other classes predicted as delamination A. However, the situation is different with delamination B, where precision equals 0.96. This indicates that other classes are falsely predicted as delamination B. The GoogLeNet CNN is tested with a subset that includes 200 samples of four classes distributed evenly, resulting in 50 samples for each class (20% of the numerical wavefield dataset). From the confusion matrix of the testing subset, shown in Figure 19, we can see that two samples from delamination A are predicted as delamination B and one sample is predicted as pristine. Table 6 shows that the recall value of the first class, delamination A, is 0.94. This means some samples of delamination A are predicted with different labels. GoogLeNet classified the delamination D class and pristine class perfectly, which can be seen in the precision and recall values in Table 4 and Figure 19. All three metrics, precision, recall, and F1 score, present high scores with an average value of 0.98. In short, the GoogLeNet CNN classifies most of the samples accurately.

#### 5.4.2. Results and Discussion of Experimental Wavefield Dataset

**Figure 20:**
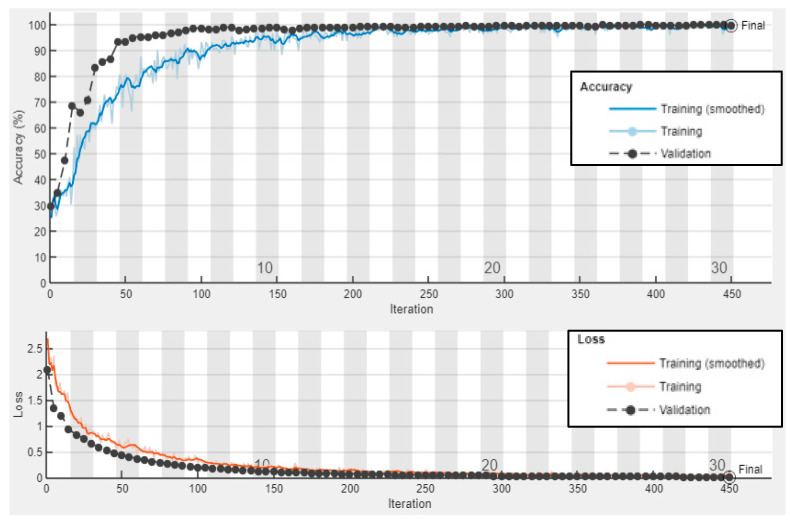
Training progress curves show the accuracy and loss varying with iteration (training process). The experimental wavefield dataset was used as an input dataset to the GoogLeNet CNN.

**Figure 21:**
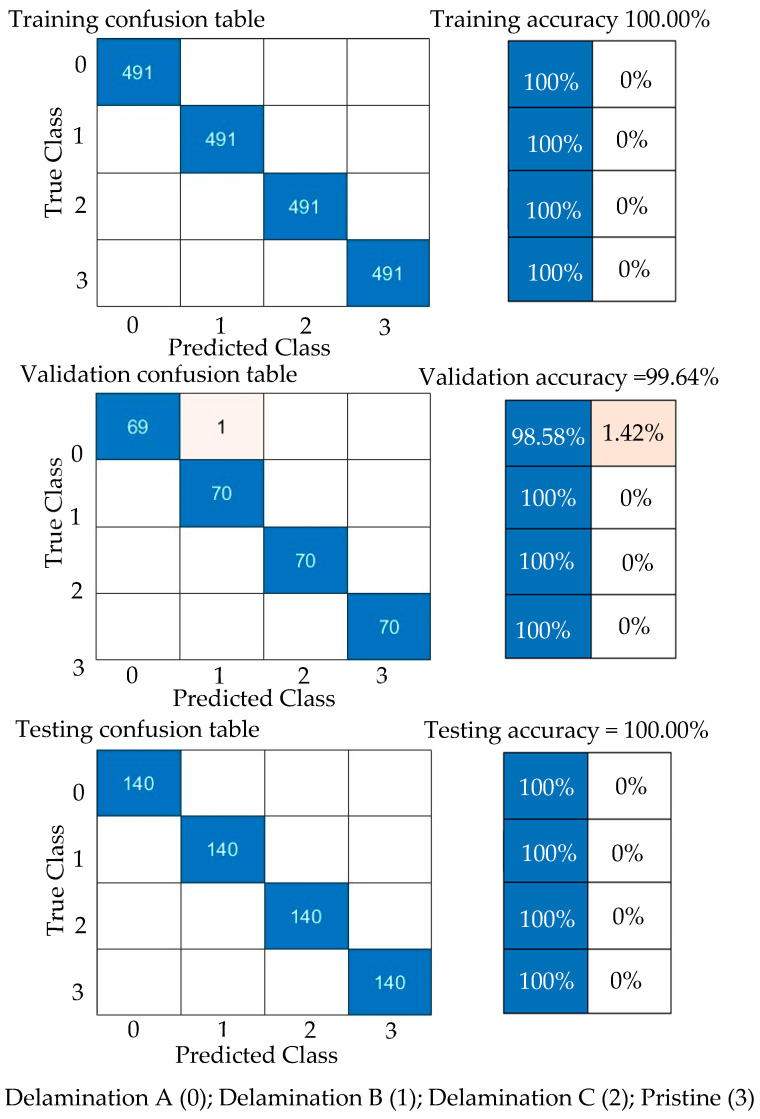
Measured Confusion matrices for training, validation, and testing GoogLeNet CNN using input experimental wavefield dataset.

Second, GoogLeNet is trained with 70% of the experimental wavefield dataset (1964 samples of four classes, 491 samples for each class). The training progress of GoogLeNet is presented in learning curves, as shown in Figure 20. It can be observed that GoogLeNet training and validation curves in both accuracy and loss plots converge at epoch 20. The GoogLeNet CNN has a training accuracy of 100%, validation accuracy of 99.6%, and testing accuracy of 100%, as seen in the confusion metrics in Figure 21. The behavior of the GoogLeNet CNN with each class using the testing experimental wavefield dataset is illustrated in the confusion matrix shown in Figure 21. This trained CNN is tested with a subset that includes 560 samples of four classes distributed evenly, resulting in 140 samples for each class (20% of the experimental wavefield dataset). It evaluates the tested dataset correctly. The values of the three evaluation metrics that describe the behavior of the CNN can be noticed in Table 7. All three metrics, precision, recall, and F1 score, present high scores with a value of 1.0. To put it briefly, all experimental samples are correctly classified by GoogLeNet.

If we make a comparison between the numerical and experimental results for both the proposed CNN model and the GoogLeNet CNN, we can observe that these CNNs have slightly low performance when using the numerical wavefield dataset. This is because of two reasons. First, the numerical dataset reflects the ideal conditions of the experiment, which, in practice, does not consider all setup parameters. For instance, the distance between the sensor and the scanning area is not considered. Second, the parameters of the experimental setup add extra features that lead to increased model capacity by accurately identifying the delamination class A, B, C, or pristine class.

#### 5.4.3. Results and Discussion of Experimental Wavenumber Spectrum Dataset

**Figure 22:**
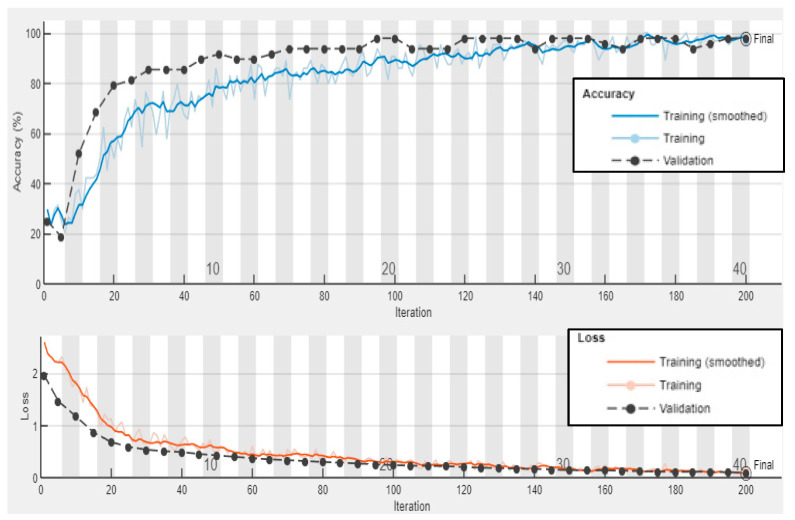
Training progress curves show the accuracy and loss varying with iteration (training process). The experimental wavenumber spectrum dataset was used as an input dataset to the GoogLeNet CNN.

**Figure 23:**
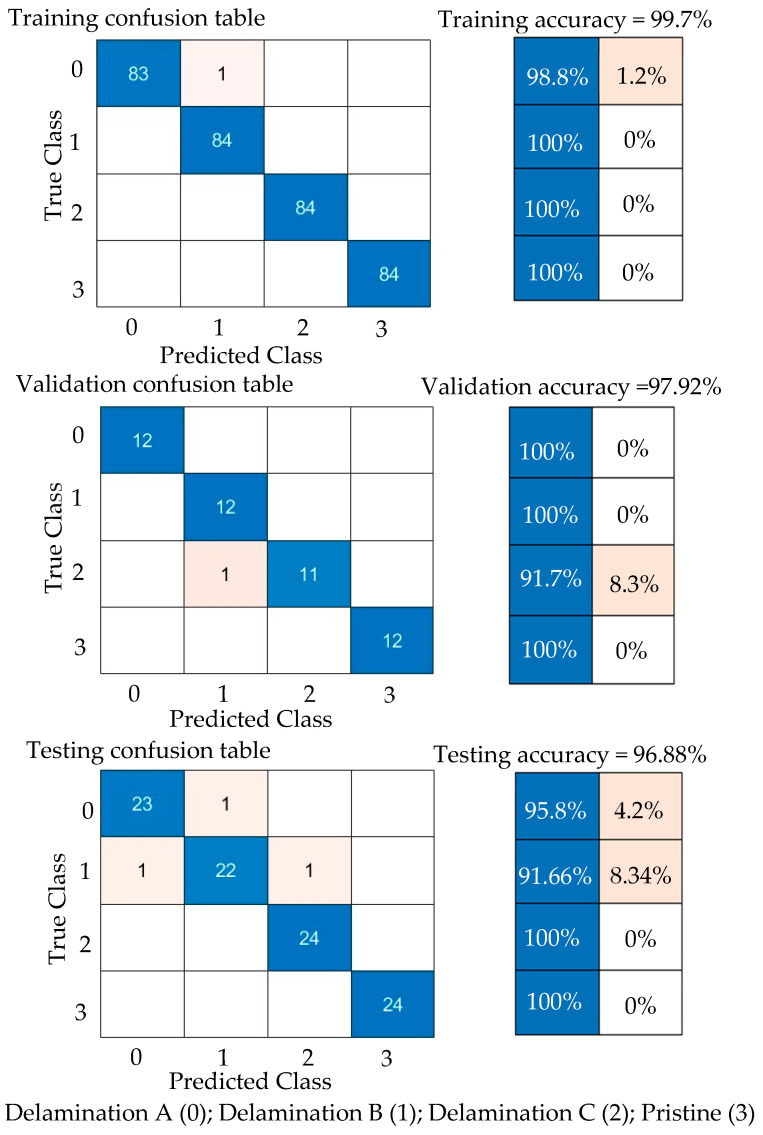
Confusion matrices for training, validation, and testing GoogLeNet CNN using the experimental wavenumber spectrum dataset as input.

Third, the experimental wavenumber spectrum dataset (480 samples of four classes) was used as input data to the GoogLeNet CNN. This CNN is trained with 70% of the experimental wavenumber dataset (336 samples of four classes, 84 samples for each class) and is validated with 10% of the experimental wavenumber dataset (48 samples of four classes, 12 samples for each class). The GoogLeNet CNN converges at epoch 40, with a training accuracy of 99.7%, and a validation accuracy of 97.92%; see the learning curves and confusion metrics in Figure 22 and Figure 23, respectively. Based on the confusion matrices presented in Figure 23, GoogLeNet shows a better performance when testing the experimental wavenumber spectrum dataset (20% of the experimental wavenumber dataset that includes 96 samples of four classes, 24 samples for each class). Table 8 shows three evaluation metrics that describe the model behavior. All three metrics, precision, recall, and F1 score, present high scores with a value of 0.97. In short, the GoogLeNet classifies most of the samples accurately.

## 6. Summary, Conclusions, and Future Work

### 6.1. Summary

This paper discussed the details of the proposed CNN model that was utilized for classifying four classes of one pristine and three different delamination classes with various depths. The input datasets to the CNN model, which were prepared using the guided wave technique, include numerical and experimental wavefield images and experimental wavenumber spectrum images. Numerical and experimental studies were conducted to observe the wavefield of Lamb wave propagation on the tested unidirectional CFRP composite plate and its interaction with three different cases of delamination. The purpose of the SLDV experiments was to validate the numerical result and to investigate the relationship between the generated trapped and scattered waves and delamination depth. The frequency–wavenumber spectrum of the time–space wavefield for pristine and delamination cases was studied experimentally. The three datasets include the numerical wavefield dataset (1004 numerical time series wavefield images), the experimental wavefield dataset (2804 experimental time series wavefield images), and the experimental wavenumber spectrum dataset (480 experimental wavenumber spectrum images). Each dataset has four equal classes of images (pristine, delamination C, delamination B, and delamination A). The proposed CNN model was applied to classify these three different datasets separately. The confusion matrix was used to present the result of classification of the training, validating, and testing sub-datasets. To test the behavior of the proposed CNN model, four metrics were used: accuracy, precision, recall, and F1 score. The results of the proposed CNN model for the three different datasets were validated using the GoogLeNet CNN.

### 6.2. Conclusions

This research presented the development of an automated system qualified for distinguishing between pristine and delamination structures and classifying three classes of delamination with various depths. This system includes a proposed CNN model and Lamb wave technique. The results show the efficacy of this system for detecting delamination depth in a CFRP composite plate. The proposed CNN model accurately classifies four classes (one pristine class and three different delamination classes) for the three independent datasets prepared from numerical and experimental SHM works. The results of the proposed CNN model present an excellent agreement with the result using the GoogLeNet CNN. The delamination depth has a significant impact on the energy level of the generated scattered and trapped waves. The results show that the generated trapped wave energy over the delamination zone decreases with increasing delamination depth and vice versa. These features (scattered and trapped waves) are utilized by the CNN to accurately classify each of the three different datasets into four classes. A significant acceptance was observed between numerical and experimental results conducted by both the proposed CNN model and GoogLeNet CNN. Trapped waves that were produced over the areas of delamination can reveal new wavenumber components in the frequency–wavenumber domain. These new wavenumber components are affected by the delamination depth. The result shows that the wavefield image and wavenumber spectrum datasets with the proposed CNN model can be used to construct a robust system with the automatic classification and detection of delamination depth. Even though the CNN model shows slightly low performance using the wavenumber spectrum dataset compared to the use of wavefield data samples, it is recommended to utilize the wavenumber spectrum as an input dataset to the CNN for several reasons. First, the wavenumber spectrum data samples are slightly affected by experimental noise compared to the image-based wavefield data samples. For this, the wavenumber spectrum dataset is preferred to train the deep learning models since they present robust feature representation. Second, the spectrum-based data samples usually involve complex patterns that can be extracted with CNN-based models. The extracted feature maps are used with clustering or classification tasks. Third, training deep learning models with the image-based wavenumber spectrum data samples often has less computation complexity compared to training the same models with time domain signals. This comes from the fact that the wavenumber spectrum data have lower dimensions that lead to reduced computation costs. Fourth, it is suitable to adopt spectrum-based data samples with CNN-based models. The visual representation presented by the wavenumber spectrum dataset can be used to highlight the main features that contribute to the classification or regression outcomes.

For improving the technology readiness level (TRL), the proposed work can be developed with two main procedures. First, additional experimental work should be conducted to collect more new data samples and form a big dataset. The big dataset would increase the CNN model capacity by considering new feature maps that help in identifying damage classes. The new dataset could include more classes. The second procedure focuses on model design. Several new deep learning techniques would be included in future work, for instance, using the YOLO algorithm to identify the conducting classes.

### 6.3. Future Work

The development of a thorough actual delamination monitoring CNN system will benefit from more studies to gather appropriate datasets of different delamination sizes to enhance categorization skills. The detection, localization, and sizing of real fatigue cracks using designed the CNN will be interesting future work.

## Figures and Tables

**Table 1:** Mechanical properties of the test unidirectional CFRP composite plate.

*E*_11_	*E*_22_	*E*_33_	*ν*_12_	*ν*_13_	*ν*_23_	*G*_12_	*G*_13_	*G*_23_	*ρ*
140.8 GPa	11.3 GPa	11.3 GPa	0.31	0.31	0.5	5.7 GPa	5.7 GPa	3.4 GPa	1640 kg/m^3^

**Table 2:** The trainable parameters for each layer of the proposed CNN model.

Model: “Sequential” Layer (Type)	Output Shape	Parameters
conv2d	(None, 222, 222, 64)	1792
max_pooling2d	(None, 111, 111, 64)	0
conv2d	(None, 109, 109, 32)	18,464
max_pooling2d	(None, 54, 54, 32)	0
dropout	(None, 54, 54, 32)	0
conv2d	(None, 52, 52, 16)	4624
max_pooling2d	(None, 26, 26, 16)	0
dropout	(None, 26, 26, 16)	0
flatten	(None, 10816)	0
dense	(None, 64)	692,288
dense	(None, 32)	2080
dense	(None, 4)	132
Total parameters: 719,380 (2.74 MB) Trainable parameters: 719,380 (2.74 MB) Non-trainable parameters: 0 (0.00 Byte)		

**Table 3:** Classification report of proposed CNN model using testing numerical wavefield dataset.

Class	Precision	Recall	F1-Score	Support
Delamination A	1.00	0.96	0.98	50
Delamination B	0.96	1.00	0.98	50
Delamination C	1.00	1.00	1.00	50
Pristine	1.00	1.00	1.00	50

**Table 4:** Classification report of the proposed CNN model using input experimental wavefield dataset.

Class	Precision	Recall	F1-Score	Support
Delamination A	1.00	1.00	1.00	140
Delamination B	1.00	1.00	1.00	140
Delamination C	1.00	1.00	1.00	140
Pristine	1.00	1.00	1.00	140

**Table 5:** Classification report of the proposed CNN model using the experimental wavenumber spectrum dataset as input.

Class	Precision	Recall	F1-Score	Support
Delamination A	0.89	1.00	0.94	24
Delamination B	1.00	0.88	0.93	24
Delamination C	1.00	1.00	1.00	24
Pristine	1.00	1.00	1.00	24

**Table 6:** Classification report of GoogLeNet CNN using the numerical wavefield dataset as input for testing.

Class	Precision	Recall	F1-Score	Support
Delamination A	1.00	0.94	0.97	50
Delamination B	0.96	0.98	0.97	50
Delamination C	0.98	1.00	0.99	50
Pristine	0.98	1.00	0.99	50

**Table 7:** Classification report of GoogLeNet CNN using the experimental wavefield dataset as input for testing.

Class	Precision	Recall	F1-Score	Support
Delamination A	1.00	1.00	1.00	140
Delamination B	1.00	1.00	1.00	140
Delamination C	1.00	1.00	1.00	140
Pristine	1.00	1.00	1.00	140

**Table 8:** Classification report of GoogLeNet CNN using testing experimental wavenumber spectrum dataset.

Class	Precision	Recall	F1-Score	Support
Delamination A	0.96	0.96	0.96	24
Delamination B	0.96	0.92	0.94	24
Delamination C	0.96	1.00	0.98	24
Pristine	1.00	1.00	1.00	24

## Data Availability

Data are contained within the article.
